# Molecular Basis of Tumor Heterogeneity in Endometrial Carcinosarcoma

**DOI:** 10.3390/cancers11070964

**Published:** 2019-07-09

**Authors:** Susanna Leskela, Belen Pérez-Mies, Juan Manuel Rosa-Rosa, Eva Cristobal, Michele Biscuola, María L. Palacios-Berraquero, SuFey Ong, Xavier Matias-Guiu Guia, José Palacios

**Affiliations:** 1 Department of Pathology, Institute Ramón y Cajal for Health Research, 28034 Madrid, Spain; 2 CIBER-ONC, Instituto de Salud Carlos III, 28029 Madrid, Spain; 3 Department of Pathology, Hospital Ramón y Cajal, 28034 Madrid, Spain; 4 Department of Pathology, Instituto de Biomedicina de Sevilla (IBiS), 41013 Seville, Spain; 5 Hospital Universitario Virgen del Rocío/CSIC/Universidad de Sevilla, 41013 Seville, Spain; 6 Department of Hematology and Hemotherapy, Clínica Universidad de Navarra, 31008 Pamplona, Spain; 7 NanoString Technologies, Inc, Seattle, WA 98109, USA; 8 Department of Pathology, Hospital U Arnau de Vilanova, 25198 Lleida, Spain; 9 Department of Pathology, Hospital U de Bellvitge, L’Hospitalet de Llobregat, 08907 Barcelona, Spain; 10 IRBLLEIDA, IDIBELL, University of Lleida, 25003 Lleida, Spain; 11 Faculty of Medicine, University of Alcalá de Henares, Alcalá de Henares, 28801 Madrid, Spain

**Keywords:** uterine carcinosarcoma, endometrial carcinoma, metaplastic carcinoma, epithelial-to-mesenchymal transition, clonality, mutation, *TP53*, PI3K/AKT pathway, gene expression, miRNA expression

## Abstract

Endometrial carcinosarcoma (ECS) represents one of the most extreme examples of tumor heterogeneity among human cancers. ECS is a clinically aggressive, high-grade, metaplastic carcinoma. At the morphological level, intratumor heterogeneity in ECS is due to an admixture of epithelial (carcinoma) and mesenchymal (sarcoma) components that can include heterologous tissues, such as skeletal muscle, cartilage, or bone. Most ECSs belong to the copy-number high serous-like molecular subtype of endometrial carcinoma, characterized by the *TP53* mutation and the frequently accompanied by a large number of gene copy-number alterations, including the amplification of important oncogenes, such as *CCNE1* and *c-MYC*. However, a proportion of cases (20%) probably represent the progression of tumors initially belonging to the copy-number low endometrioid-like molecular subtype (characterized by mutations in genes such as *PTEN*, *PI3KCA,* or *ARID1A*), after the acquisition of the *TP53* mutations. Only a few ECS belong to the microsatellite-unstable hypermutated molecular type and the *POLE*-mutated, ultramutated molecular type. A common characteristic of all ECSs is the modulation of genes involved in the epithelial to mesenchymal process. Thus, the acquisition of a mesenchymal phenotype is associated with a switch from E- to N-cadherin, the up-regulation of transcriptional repressors of E-cadherin, such as Snail Family Transcriptional Repressor 1 and 2 (SNAI1 and SNAI2), Zinc Finger E-Box Binding Homeobox 1 and 2 (ZEB1 and ZEB2), and the down-regulation, among others, of members of the miR-200 family involved in the maintenance of an epithelial phenotype. Subsequent differentiation to different types of mesenchymal tissues increases tumor heterogeneity and probably modulates clinical behavior and therapy response.

## 1. Clinicopathological Characteristics

**Figure 1:**
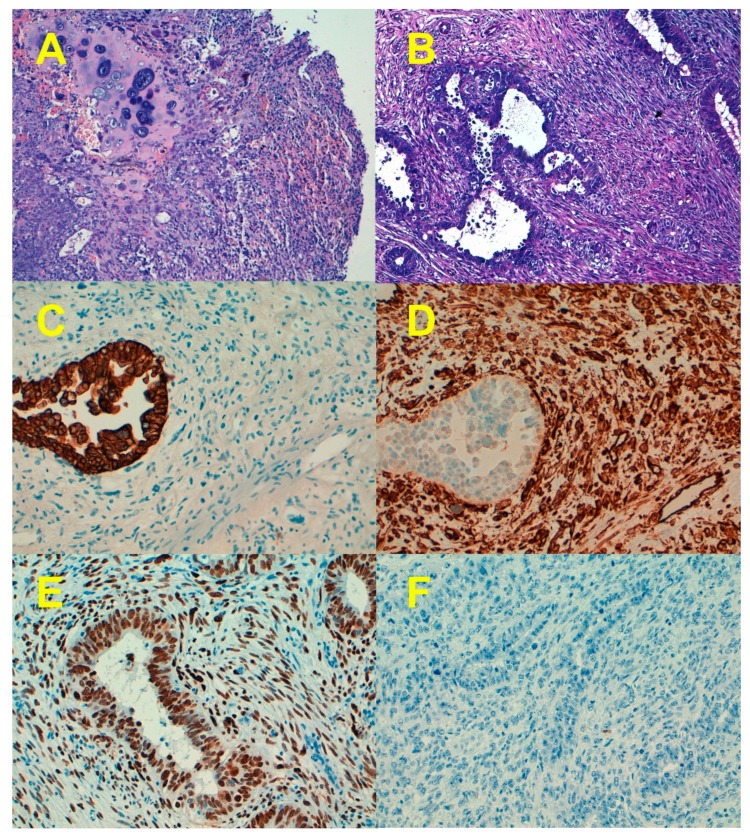
Morphological and immunohistochemical features of endometrial carcinosarcoma. (**A**) Hematoxylin-eosin staining of an endometrial carcinosarcoma showing the epithelial component surrounded by the heterologous mesenchymal component (chondrosarcoma). (**B**) Endometrial carcinosarcoma with homologous sarcoma (H&E). (**C**) Cytokeratin expression of the case depicted in b. (**D**) Vimentin expression in the case depicted in b. (**E**) p53 overexpression in both the carcinomatous and sarcomatous components. (**F**) p53 null pattern in both the carcinomatous and sarcomatous components. Only occasional normal stromal cells expressed p53. Original magnification 10× for (**A**,**B**), and 20× for (**C**–**F**).

Endometrial carcinosarcoma (ECS), also known as malignant mixed Müllerian tumor (MMMT), is a high-grade tumor characterized by a biphasic growth of malignant epithelial (carcinomatous) and mesenchymal (sarcomatous) components (Figure 1) [1]. ECS is a rare aggressive neoplasm accounting for approximately 2% to 5% of gynecological carcinomas, but it causes around 16% of all deaths due to malignancies of the uterine corpus [2,3]. Although ECS shares similar risk factors with endometrial carcinoma, such as obesity, nulliparity, smoking, and exogenous estrogen use, they present at more advanced stages and have significantly worse survival than high-grade endometrial carcinomas [3,4,5,6,7,8].

Matsuo et al. [9] analyzed the incidence of ECS in the USA during 1973–2013 in 235,849 primary endometrial carcinomas (ECs) and observed that the proportion of ECS is now significantly higher than before and accounts for more than 5% of ECs. There was a significant rise in the proportion of ECS among primary ECs from 1.7% to 5.6% during this period. Moreover, among 76,118 type II ECs, the proportion of ECS also increased significantly from 6% to 17.5%; ECS was detected in 11,000 (4.7%) women. The percentage of black women with ECS was elevated from 11.9% to 20%, whereas the proportion of white women decreased from 86% to 60.5%. The possible factors associated with the increase of ECS include the increment in the number of older women and the obese population in the US, and the global increase in the incidence of breast cancer with a concordant increment in tamoxifen use [9]. 

Several studies have demonstrated that tamoxifen use may be associated with an increased incidence of ECS. In women with breast cancer, the incidence of ECS is 6.35-fold higher in those treated with tamoxifen [10]. Matsuo et al. [11] reported that ~6% of women with ECS have a history of tamoxifen use and that tamoxifen-related ECS was significantly associated with a higher proportion of stage IA disease (48.4% versus 29.9%) and a lower risk of stage IVB disease (7.8% versus 16%) compared to tamoxifen-unrelated ECS. Deep myometrial tumor invasion was less common in uterine carcinosarcoma related to tamoxifen use (28.3% versus 48.8%). However, in spite of these favorable tumor characteristics, tamoxifen-related ECS had comparable stage-specific survival outcomes compared to tamoxifen-unrelated ECS.

**Figure 2:**
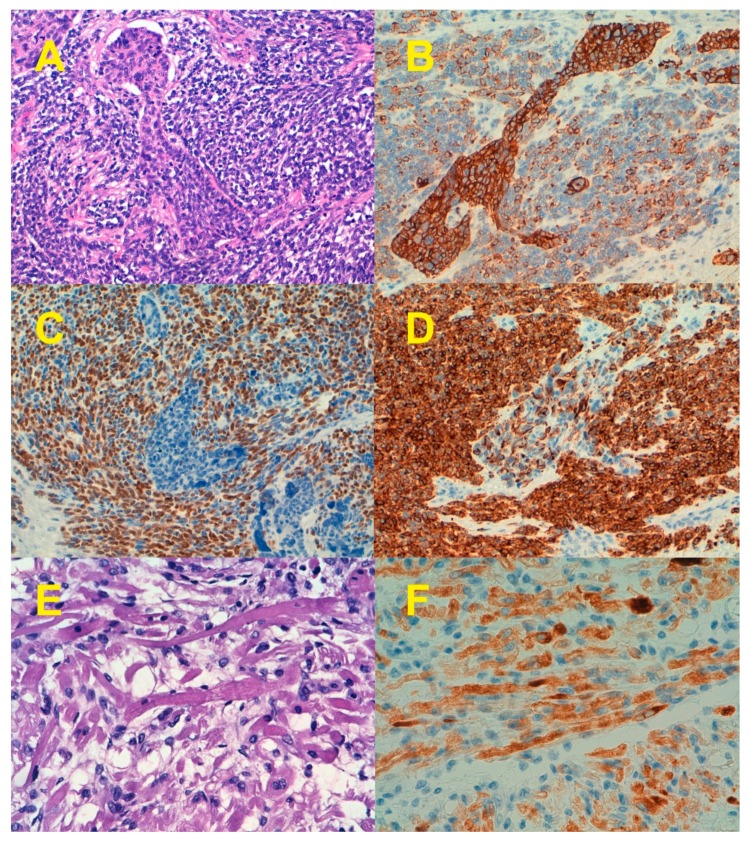
Endometrial carcinosarcoma with rhabdomyoblastic differentiation. Some cells showed an intermediate epithelial/mesenchymal differentiation as suggested by the expression pattern of cytokeratins, myogenin, and desmin. (**A**) Hematoxylin-eosin staining. (**B**) Cytokeratin (CK AE1/AE3) expression. (**C**) Myoblast determination protein 1 (MyoD1) expression. (**D**) Desmin expression. (**E**) Striated rhadbomyoblasts (H&E). (**F**) Desmin expression by striated rhadbomyoblasts. Original magnification 20× for A-D, and 40× for E and F.

From a morphological point of view, the epithelial component of ECS could be endometrioid (most common in most series) or non-endometrioid (serous, clear cell, undifferentiated, or mixed) [3,4,12,13,14,15]. Matsuo et al. [16] reported that among 906 ECS evaluated for histological patterns in their series, high-grade carcinoma/homologous sarcoma (40.8%) was the most common type followed by high-grade carcinoma/heterologous sarcoma (30.9%), low-grade carcinoma/homologous sarcoma (18%), and low-grade-carcinoma/heterologous sarcoma (10.3%). In 75% to 95% of ECS, the epithelial component was of high grade [16,17]. The mesenchymal component could be minimal or extensive. Sarcoma dominance (SD) is defined by the presence of more than 50% of the tumor composed by the sarcomatous component. The mesenchymal component could be subdivided into homologous (fibrosarcoma, leiomyosarcoma, and endometrial stromal sarcoma) and heterologous, the latter including skeletal muscle, cartilage, fat, or osteoid, which is present in up to 60% of tumors [3,4,12,13,14,15,16,17,18,19]. Immunohistochemistry may be useful in confirming the presence of a heterologous mesenchymal component, which, as discussed later, is an adverse prognostic indicator in some series. For example, nuclear staining with myogenin and Myoblast determination protein 1 (myoD1) helps to confirm the presence of rhabdomyoblastic differentiation (Figure 2) [20].

Regarding other pathological features, 55% to 60% of ECS show less than 50% of myometrial invasion at diagnosis. Lymphovascular invasion (LVI) prevalence in ECS seems to be higher than in other types of endometrial cancer (60.4–62% vs. 26–52%) [21]. Matsuo et al. [21] reported that among LVI-positive cases, LVIs with a carcinomatous component alone was found in 76.8% and LVI containing a sarcomatous component with or without a carcinomatous component in the remaining 23.2%. Tumors in the LVI-sarcoma group were more likely to have SD (82.1% vs. 26.4%), heterologous sarcomatous component (51.3% vs. 37.9%), low-grade carcinoma (42.5% vs. 22.4%), and large tumor size (81% vs. 70.2%) in the primary tumor site compared with tumors in the LVI-carcinoma group.

Also, the pattern of metastasis differs between the epithelial and mesenchymal parts of the ECS. Thus, for example, Matsuo et al. [16] analyzed 1096 metastatic sites and showed that carcinoma components tended to spread lymphatically, while sarcoma components tended to spread locoregionally (cervix, vagina, etc.).

ECS follows an aggressive clinical course. Patients with International Federation of Gynecology and Obstetrics (FIGO) stage 1–2 disease have a five-year disease-specific survival of 59%, while those with stage 3 and 4 disease have a five-year disease-specific survival of 22% and 9%, respectively [2]. The most important prognostic factors in these tumors include FIGO stage and depth of myometrial invasion [5,7,8,13,15,22]. Other known clinicopathologic features associated with worse outcome are the grade and histology of the epithelial component and lymphovascular invasion [3,5,8,13]. Although the grade and the amount of the sarcomatous component and the presence of heterologous elements are not related to the overall outcome in some series [6,13,18,22], recent studies have shown the importance of the sarcomatous component in the prognosis and response to radiotherapy [17,23]. Thus, Matsuo et al. [24] reported that ECS with better prognosis were those composed of a low-grade carcinoma and homologous sarcoma without SD. In contrast, the worse prognosis corresponded to ECS composed of a high-grade carcinoma and heterologous sarcoma and SD. This latter type of tumor tended to occur in older, obese, and Caucasian patients, and they were more likely to have metastatic implants, large tumor sizes, LVI with sarcoma cells, and higher lymph node ratios. Also, SD seems to be a prognostic factor in some series [17,23], and it is associated with loco-regional tumor metastasis and recurrence with sarcoma. In addition, ECS with SD seems more sensitive to radiotherapy compared to ECS without sarcoma dominance [23]. Finally, different studies have reported a poor prognosis in ECS with rhabdomyoblastic differentiation [4,17,19]. 

Molecular studies have demonstrated similar genetic alterations in both the carcinomatous and sarcomatous components of ECS (Table 1). Thus, it is now accepted that most carcinosarcomas are in fact metaplastic carcinomas, in which the sarcomatous component is derived from the carcinomatous component as a result of transdifferentiation (epithelial-to-mesenchymal transition—EMT) during the evolution of the tumor as shown in several studies [25,26]. However, a small percentage of ECS probably represent real collision tumors, since they are molecularly biclonal and most likely develop from two independent cell populations [6,27]. 

## 2. Molecular Subtypes of ECS

Four molecular groups have been defined for ECs: the hypermutated (mismatch repair deficiency), the ultramutated (*POLE* mutated), the copy-number low, and the copy-number high groups. These groups not only have different molecular alterations but also different prognoses; patients from the ultramutated group show the best prognosis, whereas patients in the copy-number high group have the highest risk of recurrence [28]. 

Considering the mutational profile (Table 1; see below Section 2. Molecular Subtypes of ECS), most ECSs are similar to serous-like, copy-number high ECs. Thus, in the study by McConechy et al., most tumors had a molecular profile similar to endometrial serous carcinoma (characterized by the presence of *TP53*, *FBXW7,* and *PPP2R1A* mutations and the absence of *ARID1A*, *CTNNB1*, *KRAS,* or *PTEN* mutations), while part of the tumors displayed an endometrioid carcinoma-like mutation profile characterized by the presence of *ARID1A*, *CTNNB1*, *KRAS,* and *PTEN* mutations. Based on both combined genetic and immunohistochemical profiles in their cohort, 18 tumors presented serous-like and 11 tumors presented endometrioid-like molecular profiles. There was a good correlation between the histological subtyping (taking into account the morphology of the epithelial component) and the molecular subtyping in 27 of 29 uterine carcinosarcomas (93%) [29]. More recently Jones et al., applied this classification to their set of tumors, and were able to classify 55 out of 57 tumors, of which 22% were endometrioid and 78% serous-like ECS. One sample did not fit in the model due to an ultramutated phenotype caused by the *POLE* mutation, while another had no mutation in the genes used for classification. Interestingly, all 10 stage IV tumors were serous-like [30].

Most of the endometrioid-like ECSs also showed *TP53* mutations, implying that *TP53* could be involved in the progression of part of the copy-number low endometrioid-like carcinomas to ECSs, as we have previously reported in undifferentiated endometrial carcinoma [31]. Very few ECS belong to the microsatellite-unstable hypermutated molecular type and the *POLE*-mutated ultramutated molecular type used for the classification of endometrial carcinoma. The molecular heterogeneity present in ECS opens opportunities for targeted therapies.

## 3. Serous-Like Molecular Alterations in ECS

Previous studies combining aberrant expression of p53 and mutational analysis estimated a *TP53* mutation prevalence of 50–60% [3,12,22,27,32,33,34,35]. However, subsequent studies using Next Generation Sequencing (NGS) techniques have shown that the true frequency of *TP53* mutation in ECS is very high, between 64% and 91% [29,30,36,37,38,39,40]. In effect, *TP53* mutations are the most frequent molecular alterations in ECS (Table 1). The lack of nuclear p53 expression is most commonly detected with indel or nonsense mutations, while missense mutations usually lead to diffuse nuclear p53 immunostaining. Most of the mutations are located in the DNA binding domain, and very few are present in the translocation and tetramerization motifs. In the DNA binding domain, 32% of mutations are located on known hotspot residues, and the most frequent are the R248Q and R273C/H (12% and 7%, respectively) followed by H179R/D, H193R/Y, and S241Y (5% each), (http://cancergenome.nih.gov/) [41]. 

The carcinomatous and sarcomatous components show a concordance of 85% for the p53 protein overexpression and 96% for the *TP53* gene mutation, which points to a monoclonal origin of both components (Figure 1). p16 overexpression (in-block diffuse expression) occurs in about 60% of ECS simultaneously with TP53 mutations. The concordance of p16 expression between the carcinomatous and sarcomatous components was about 85% in different series [12,35,42,43,44]. In addition to *TP53*, ECSs show mutations in other genes that are also more frequently affected in endometrial serous carcinoma (ESC) than in endometrial endometrioid carcinoma (EEC). Accordingly, mutations of *FBXW7* and *PPP2R1A* have been reported in 19% to 39% and 1% to 38%, respectively, in different series [36,37,38,39,40]. 

Regarding the *BRCA1* and *BRCA2* genes, the frequency of ECS in patients carrying germinal *BRCA1/2* mutations has been analyzed in different studies. The estimated relative risk for mutation carriers is approximately 2% per year, most importantly among serous carcinoma [45,46,47]. A recent series has reported that *BRCA1/2* were found mutated in 18% and 27%, respectively of ECS [30], although in the TCGA (The Cancer Genome Atlas Program) series, only *BRCA2* mutations were detected and at a lower frequency (5%) [28]. Carcinosarcoma of the breast and ovary have been reported in some patients with *BRCA1/2* germline mutations [48,49,50].

Zhao et al. [51] found an excess of mutations in genes encoding *histone H2A* and *H2B*, as well as a significant amplification of the segment of chromosome 6p harboring the histone gene cluster containing these genes. Thus, mutations in histone *H2A/H2B* genes were significantly enriched in carcinosarcomas (CSs) compared with carcinomas (mutations in 21.2% of CSs and 5.2% of uterine and ovarian epithelial tumor). These findings implicate mutations in histone *H2A/H2B* genes in ECS. 

Le Gallo et al. [40] have reported forkhead box A2 (*FOXA2*) mutations in 15.1% of ECS. *FOXA2* had not previously been implicated in ECSs and was predominated by frameshift and nonsense mutations. Sequencing of *FOXA2* in 160 primary endometrial carcinomas revealed somatic mutations in 5.7% of serous, 22.7% of clear cell, 9% of endometrioid, and 11.1% of mixed endometrial carcinomas, the majority of which were frameshift mutations. Collectively, the findings of the study of Le Gallo et al. [40] provide evidence that *FOXA2* is a pathogenic driver gene in the etiology of primary uterine cancers, including ECSs.

Similarly to ESC, ECS is characterized by aneuploidy and a high frequency of copy number variations (CNVs). Analysis of ploidy and whole-genome doubling has established a median ploidy of 3.3 and that 90% of ECS had undergone at least one whole-genome-doubling event. This percentage is significantly higher than in serous ovarian tumors, the tumor type with the next highest frequency of genomic doubling in the TCGA [38]. 

**Figure 3:**
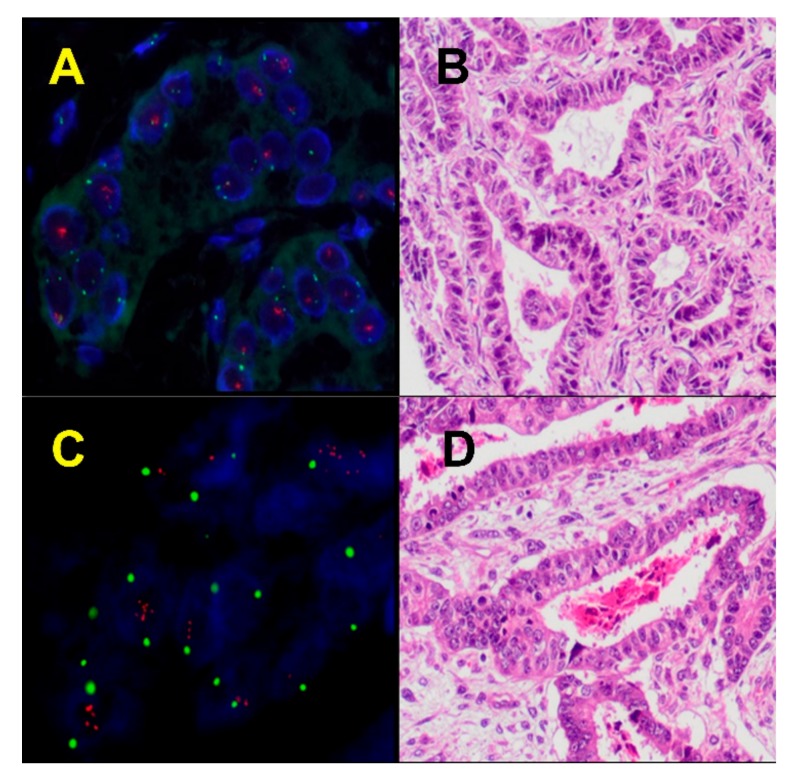
Amplification of oncogenes in endometrial carcinosarcomas analyzed by fluorescence in situ hybridization (FISH) (**A**) and (**B**), MYC proto-oncogene, bHLH transcription factor (*MYC*) amplification (**C**), and (**D**) Cyclin D1 (*CCND1*) amplification. Original magnification ×100 for A and C, and ×20 for B-D.

Recurring focal amplifications reported in the TCGA [38], some of which have also been observed in other series [51], include those containing known oncogenes such as *TERC* (3q26.2), *FGFR3* (4p16.3), *MYC* (8q24.21), *KAT6A* (10q22.2), *MDM2* (12q15), *ERBB2* (17q12), CCND1 (11q13), *CCNE1* (19q12), *BCL2L1* (20q11.21), and *RIT1* (1q22) (Figure 3).

Cyclin D1 (*CCNE1*) is the most frequently amplified gene in ECS, 41% according to data derived from TCGA (Table 1). In other tumors, for example, ovarian high-grade serous carcinoma, amplification of *CCNE1* is associated with a worse prognosss and resistance to chemotherapy. According to Schipf et al., *c-MYC* amplification had a higher frequency in the carcinomatous compared to the sarcomatous tumor component. In their data on 30 carcinosarcomas of the ovary and uterus, *c-MYC* gene amplification was reported in 78% by fluorescence in situ hybridization (FISH) [52]. However, the TCGA data showed amplification of *c-MYC* in only 21% of ECSs [38].

The frequency of *ERBB2* amplification in ECS ranged from 3–20% [30,38,53,54,55]. Thus, ECS patients with *ERBB2* amplification could benefit from anti-HER2 (human epidermal growth factor receptor 2) therapies, such as Trastuzumab. For patients unresponsive to chemotherapy and Trastuzumab, T-DM1 (Trastuzumab emtansine) may offer an alternative treatment option, as recent studies show how ECS cell lines and derived xenografts with *ERBB2* amplification respond well to T-DM1 [56]. *PIK3CA* is amplified in 11% of ECS, further highlighting the importance of the phosphatidylinositol 3-kinase (PIK3) pathway (see Section 4. Endometrioid-Like Molecular Alterations). 

Schipf et al. detected *ZNF217* amplification in 87% of gynecological CS [52]. Similarly to *c-MYC*, in the TCGA data set the frequency is much lower (9%) [28,38]. Two other frequently amplified oncogenes in ECS, *EGFR,* and *URI* (unconventional prefolding RPB5 interactor 1), have not been found in the TCGA data set. Biscuola et al. reported *EGFR* amplification by FISH in 19% of tumors [57], while in studies with smaller sample size, EGFR (epidermal growth factor receptor) protein overexpression has been reported in 45% to 82% of ECS, where a higher level of expression was seen in the sarcomatous component [53,58,59]. *URI1* amplification has been reported in 40% of ECS [60]. *URI1* amplification was also associated with poor survival and reduced response to adjuvant treatment. Likewise, in a cultured cell model, overexpression of *URI1* induced *ATM* (ATM Serine/Threonine Kinase) expression and resistance to cisplatin [60]. Recurring *GPC5* (Glypican 5) gain/amplification has been detected in a subset of ECS, mostly in the sarcoma component, and the authors linked the involvement of *GPC5* with sarcomatous transformation [61].

## 4. Endometrioid-Like Molecular Alterations

Mutations in genes encoding for the kinase or regulatory proteins of the PI3K/AKT (phosphatidylinositol 3-kinase/(Protein Kinase B) pathway have been detected in up to 67% of ECS [29]. Moreover, multiple PI3K/AKT pathway proteins have been found mutated in one tumor. *PIK3CA* mutations have been found in 11% to 40% [29,30,36,38,57,62,63] of the tumors. Unlike for *TP53*, with mutations concentrated on HotSpot regions, the mutations in *PIK3CA* are found scattered in the different functional domains. In addition to the traditional *PIK3CA* hotspots in exons 9 and 20, a smaller portion of ECS has mutations in exon 1, in the adaptor binding domain, helical domain, and C2 domain which increase kinase enzymatic activity [29,57].

The importance of mutations in this pathway comes from the fact that *PI3KCA* mutations have been detected in both the carcinoma and sarcoma components of the primary tumor and also in the metastatic tumor. This implies that they are important early events in the tumorigenesis of carcinosarcoma and thus could be targeted with PIK3CA/mTOR (Phosphatidylinositol-4,5-Bisphosphate 3-Kinase Catalytic Subunit Alpha /Mechanistic Target of Rapamycin Kinase) inhibitors [29,38]. PIK3CA inhibition has been applied successfully in advanced endometrial cancers [64].

Phosphatase And Tensin Homolog (*PTEN)* mutations are not as frequent as *PIK3CA*, but they are present in approximately 20% of ECS: 17% and 19% in the series reported by McConechy et al. [29] and the TCGA [38], respectively. However, Jones et al. reported that 47% of ECS carried *PTEN* mutation, but their series included only 17 cases [36]. *PTEN* and *PIK3CA* mutations frequently coexist in the same ECS [29]. 

Other genes with less frequency of mutations in the PI3K/AKT pathway in ECSs include Phosphatidylinositol 3-Kinase Regulatory Subunit 1 (*PIK3R1)* (10–17%), *PIK3R2*, *AKT1*, *AKT2,* and *AKT3* (less than 5% for each gene) [29,36,38,57].

AT-Rich Interaction Domain 1A (*ARID1A)* and Catenin Beta 1 (*CTNNB1)* are commonly mutated in EEC, and *ARID1A* mutations occur also in 10% to 15% of ECS, leading usually to loss of of protein expression, while mutations in *CTNNB1* are infrequent in ECS [36,38,63]. *KRAS* mutations were found in 12% and Cadherin 4 (*CDH4)* mutations in 18% [38]. 

Mismatch repair deficiency (MMR-def) and *POLE* mutations are more common in EEC than in ESC. MMR-def is due to germline or somatic even affecting mismatch repair genes, most frequently MutL Homolog 1 (*MLH1*), MutS Homolog 2 (*MSH2*), MutS Homolog 6 (*MSH6*)*,* and Mismatch Repair Endonuclease PMS2 (*PMS2*). In sporadic EC, MMR-def is detected in 15–30% of cases [65], although a higher frequency has been detected among high-grade endometrioid carcinomas (45–63%) [31], most frequently due to *MLH1* promotor methylation. In addition, between 2–6% of endometrial carcinoma occurs in the context of Lynch syndrome due to germline mutations [66]. The frequency of MMR-def varies between 3% and 23% in ECS. The higher frequencies come from studies with a small sample size [36,67], while lower percentages have been observed in a bigger series [37,68]. *MLH1* promoter methylation is probably the major cause for MMR-def in most tumors [68], and accordingly, *MLH1* was epigenetically silenced in the two samples with MMR-def in the TCGA series [38]. 

Mutations in DNA Polymerase Epsilon, Catalytic Subunit *(POLE*) are present in some ECS, both of the most common HotSpot-mutations (P286R and V114L) have been identified in individual cases of ECS [38,69,70]. The most common mutations detected by NGS in recent studies are shown in Table 2.

## 5. Gene Expression Profiles in ECS

Several studies have analyzed mRNA and microRNA (miRNA) expression profiles in ECS in comparison to other histological types of EC [71]. Regarding mRNA expression profiles, ECS differs from other EC histotypes in the expression, among others, of genes modulating epithelial-to-mesenchymal transition (EMT) and immune response (see Section 9. Immune Response in CS), and in the expression of cancer-testis antigens (CTA).

There are over 200 CTAs, which are classified into different families according to their sequence homology. In general, CTA genes are expressed only in normal testis and cancerous tissue. In many instances, CTA families are formed by clusters of nearly identical genes that are frequently located on the X-chromosome. A shared regulatory mechanism for related CTA clusters has been suggested as whole families of CTAs are often co-expressed together in tumors [72,73].

Overexpression of many members of the CTA family, such as melanoma antigen family A (*MAGEA*) members (*MAGEA6*, *MAGEA9*, *MAGEA12*), *XAGE2*, *CTCFL*, and *CTAG1A* (cancer/testis antigen 1A) has been reported in ECS [73]. *CTCF*, also known as the brother of the regulator of imprinted sites (*BORIS*), is an oncogene that deregulates the cancer epigenome, which is a common event in ECS [73,74]. Expression of CCCTC-Binding Factor Like (*CTCFL*) probably mediates the demethylation of another CTA gene, thus resulting in activation via repression [74]. Other genes of the CTA family associated with ECS, include, New York esophageal squamous cell carcinoma-1 (*NY-ESO-1*) and Preferentially Expressed Antigen In Melanoma (*PRAME*) [75,76]. Considering the tissue-restricted expression of CTA and its immunogenicity, immunotherapy based on CTA vaccines might be beneficial to ECS patients [73].

The miRNA signature of carcinosarcomas differs from endometrioid and serous carcinomas [77]. The function of miRNAs is to regulate gene expression by silencing. For this, they pair to the three prime untranslated region (3’UTR) of the target mRNA sequence and thereby direct their posttranscriptional repression. miRNAs are small noncoding RNAs, which in turn can be regulated by promotor methylation and transcription factors, or by miRNA processing and stability [78].

In addition to miRNAs related to EMT (see Section 7. Epithelial-to-Mesenchymal Transition), miR-20b, miR-301, and miR-487 are up-regulated in carcinosarcomas compared to both endometrioid and serous tumors, whereas miR-518b is down-regulated. Low expression of miR-20b seems to inhibit tumor cell growth but then again help the tumor cell to gain resistance to apoptosis in hypoxic conditions [79]. In another study, miR-888 overexpression was detected in ECS, and the progesterone receptor was its direct target [80]. Finally, lower cancer-specific survival has been associated with upregulation of miR-184 and downregulation of let-7b-5p and miR-124 [81].

## 6. Methylation Profiles in ECS

Similarly to other types of cancers, ECS displays abnormal DNA methylation patterns including genome-wide hypomethylation and site-specific hypermethylation, associated with increased expression of DNA methyltransferases (DNMT1, DNMT3a), when compared to the normal endometrium [38,82]. Regarding global hypomethylation, Li et al. [82] reported that in normal endometrium, the 80% of analyzed CpGs were methylated, whereas, in ECS samples, this ratio fell to 60% to 70%. In addition, all major classes of genomic transposable elements exhibited global DNA hypomethylation in ECS, with Long interspersed nuclear elements (LINEs) exhibiting the largest effect size. This effect was greater in ECS than in other histological types of endometrial carcinomas.

A number of tumor suppressor genes with recurrent hypermethylated promoters has also been reported in ECS, *KLF4*, *NDN*, *WT1*, *PROX1*, among others. Promoter hypermethylation of these genes is also common in other types of EC [38,82]. Interestingly, Cherniak et al. [38] reported that unsupervised cluster analysis of DNA methylation profiles of ECS grouped the tumors into three main classes according to their cancer-specific hypermethylation patterns. One group of tumors exhibited a hypermethylation pattern similar to that of EEC, whereas the others were much more similar to the ESC. Accordingly, the frequency of *PTEN* mutations was higher in the first group. 

A constant characteristic of ECS is the aberrant DNA methylation of miR-200 genes (see discussion in Section 7. Epithelial-to-Mesenchymal Transition).

## 7. Epithelial-to-Mesenchymal Transition

EMT is a biological process that involves the acquisition of a mesenchymal/stem-cell-like phenotype by the (malignant) epithelial cells, endowing these cells with migratory and invasive properties, promoting cancer progression, preventing cell death and senescence, and inducing resistance to chemotherapy [83]. EMT has an important role in cancer, especially in tumor invasion and metastasis. During EMT, epithelial cells undergo a ‘‘cadherin switch’’ in which expression of N-cadherin is increased and E-cadherin expression reduced. E-cadherin can be repressed by either zinc-finger transcription factors (Snail1 (SNAI1), Slug/Snail2 (SNAI2), ZEB2 (SIP1) and ZEB1 (δ-EF1)) or basic helix–loop–helix transcription factors (E47 (TCF3), E2-2 (TCF4) or Twist). These EMT transcription factors (EMT-TF) can become activated through activation of different pathways such as Transforming Growth Factor Beta 1 (TGFβ), tyrosine kinase receptors and Wnt, among others [25].

We have previously suggested that EMT is activated in ECS [65,73,84,85]. Further studies have confirmed this suggestion [25,38,39,84,86]. For example, we used real-time PCR to measure the differences in the expression of, E-cadherin, cadherin-11, *SPARC, SNAIL*, *ZEB1*, *ZEB2*, *TWIST-1*, *TCF4*, *TGFβ1,* and *TGFβ2* between the epithelial and mesenchymal components of 23 ECSs. Also, we used immunohistochemistry to evaluate the expression of E-, P- and N-cadherin, cadherin-11, p120, vimentin, SPARC, fascin, and caveolin-1 in 76 ECS. In the mesenchymal component, a ‘‘cadherin switch’’ from E-cadherin to N-cadherin and cadherin 11 was observed. In addition, upregulation of all of E-cadherin repressors together with overexpression of all mesenchymal markers tested was demonstrated.

Also, High Mobility Group AT-Hook 2 (HMGA2) has a role in EMT as a regulator of SNAI1 expression and of other transcription factors downstream of SNAI1, such as Slug, ZEB1, and ZEB2. HMGA2 has been proposed to be regulated by the let-7/Lin28B pathway. Accordingly, we have previously demonstrated that an increase of Lin28B expression correlated with let-7b down-regulation and HMGA2 overexpression in ECS [73]. 

A role of the WNT pathway in the transition from an epithelial to a mesenchymal status is demonstrated by the fact that up to 23% of ECS showed nuclear β-catenin, not associated with *CTNNB1* mutation, in the sarcomatous but not in the carcinomatous component [57]. Nuclear β-catenin cooperates with Sox4 and p300 to transcriptionally up-regulate Slug to induce EMT [87].

Similarly to β-catenin, in another study, ALK tyrosine kinase receptor (ALK) was frequently over-expressed in the sarcomatous components of EC [87]. The authors suggest that ALK-related cascades could participate in divergent sarcomatous differentiation through the induction of EMT and inhibition of apoptosis [87]. In contrast, although the expression of L1CAM is a strong predictor of poor outcome in endometrial cancer and overexpression of L1CAM has been related to EMT in endometrial cancer cell lines [88], in clinical samples of ECS, only the epithelial component was positive in 65% of the cases, while no expression was seen in the mesenchymal part. Thus in ECS, L1CAM is not a marker for the mesenchymal phenotype [89].

MicroRNA signatures associated with EMT and their relationships with EMT markers in human carcinosarcomas have been studied by us and more recently by Cherniack et al. [25,38,84]. We used real-time PCR to measure the differences in the expression of 384 miRNAs, between the epithelial and mesenchymal components of ECS and found that miR-200 family members were down-regulated in the mesenchymal part of the ECS. The miR-200 family plays a major role in regulating epithelial plasticity, mainly through its involvement in double-negative feedback loops with the EMT-TFs ZEB1, ZEB2, SNAI1, and SNAI2, ultimately influencing E-cadherin expression levels [25,84,85]. Down-regulation of miR-200 family members in ECs is not only due to the transcriptional repression by EMT-TF, but also to promoter methylation [38,84]. In this sense, experimental studies have demonstrated a major role of ZEB1 in transcriptional repression and of SNAI1 and, to a lesser extent, SNAI2 in the epigenetic silencing through DNA hypermethylation of miR-200 genes [84]. Other down-regulated miRNAs in our studies included miR-23b and miR-29c, involved in the inhibition of mesenchymal markers, and miR-203 and miR-205 involved in the inhibition of cell stemness [25,84].

## 8. Beyond EMT: Stemness and Differentiation in ECS

It has been demonstrated that epithelial cells undergoing EMT to acquire mesenchymal features are more likely to possess stemness. In addition, some studies suggested that stemness can be associated with cells undergoing a partial EMT and showing a hybrid Epithelial/Mesenchymal phenotype. Jolly et al. postulated that the core EMT and stemness modules, miR-200/ZEB and Lin28/let7, govern EMT decision making [90]. According to this hypothesis, not only the miR-200/ZEB EMT module is active in ECS, as previously discussed, but also, we have previously demonstrated that the expression of the suppressor of miRNA biogenesis Lin28B was increased in ECS when compared with EEC samples (62.85-fold change). Moreover, we observed a significant inverse correlation between the expression of Lin28B and let-7b, supporting the hypothesis that they participate in the same regulatory pathway [73].

Cells with an Epithelial/Mesenchymal hybrid phenotype evolve to an epithelial or a mesenchymal phenotype depending on factors acting on the EMT and stemness modules [91]. Both routes would enable a secondary round of differentiation to specific epithelial or mesenchymal phenotypes [92]. ECS exemplified well this hypothesis since different types of mesenchymal tissues could develop. This is illustrated not only by the morphological evidence of striated muscle, cartilage, or bone tissue in ECS but also by molecular evidence. Thus, the presence of rhabdomyoblastic differentiation in ECS, the most common heterologous mesenchymal differentiation in ECS, is accompanied by the overexpression of genes that are characteristic of primary embryonic myocytes [93]. Romero-Perez et al. [73] demonstrated that in ECS there was an overexpression of the core network of transcription factors that control the myogenic program in primary myocytes, including Myf5, Myf6, MyoD, and MYOG (myogenin), in addition to other transcriptional factors involved in this process, such as SIX1 and EYE1/2. Moreover, overexpression of genes encoding specialized cytoskeletal proteins, such as slow (*Myh7*) and embryonic (*Myh3*) myosin heavy chains and skeletal α-actin (*Acta1*), was also observed. Similar to our previous results, Lu et al. [94] reported that 18 out of 57 ECS reported in the TCGA had a gene expression pattern enriched in genes involved in muscle development and morphogenesis, myoblast differentiation, and contraction regulation.

## 9. Immune Response in CS 

The tumor microenvironment has an important role in cancer and immunomodulation of the microenvironment is a new focus in cancer medicine [95]. Accumulated evidence indicates that ECS is a rational target for immune therapy. In their study of gene expression, Romero-Peréz et al. found that over 10% of the genes differentially expressed between ECS and EEC were implicated in the immune response, suggesting differential immunomodulation between histotypes [73]. 

**Figure 4:**
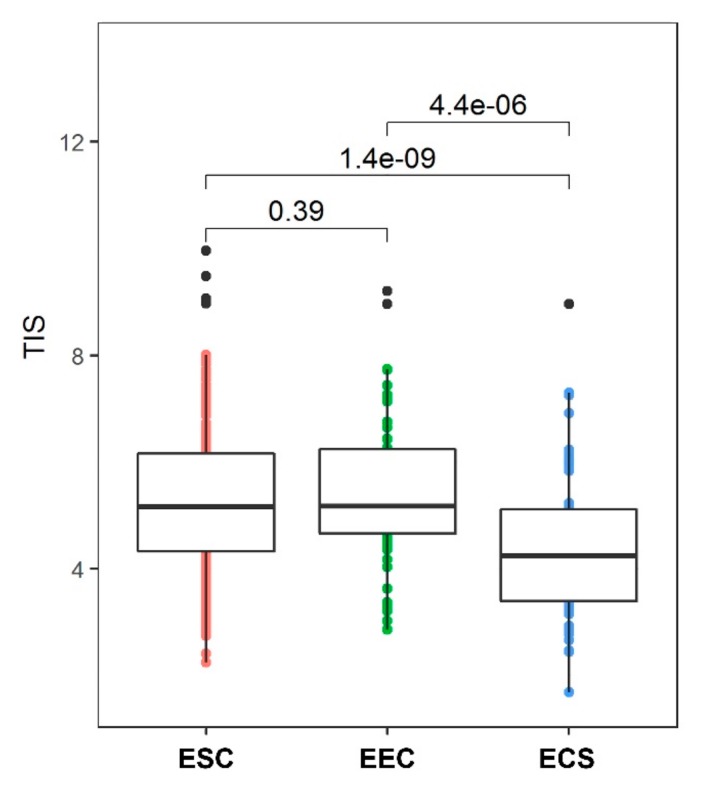
A boxplot histogram of Tumor Inflammation Signature (TIS) scores by endometrial cancer type in endometrial carcinosarcoma (ECS), endometrial serous carcinoma (ESC), and endometrial endometrioid carcinoma (EEC). *p* values from analysis of variance (ANOVA) test are shown for all comparisons.

Ayers et al. have created a Tumor Inflammation Signature (TIS) using gene expression data from baseline tumor samples of pembrolizumab-treated patients. The signature includes 18 genes that reflect a suppressed adaptive immune response (antigen presentation, chemokine expression, cytotoxic activity, and adaptive immune resistance) and is enriched in tumors with sensitivity to Programmed cell death 1 ligand 1 (PD-L1) inhibitors [96]. In another study, Danaher et al. concluded that, although there was only a correlation between TIS and tumor mutational burden (TMB), the tumors could be classified equally well with either TIS or TMB [97]. Using data from TCGA, we compared the TIS between endometrioid and serous endometrial and ECS and observed that it is significantly lower in uterine carcinosarcoma ECS compared to both ECS and ESC (analysis of variance (ANOVA), p < 0.001, Figure 4). However, TIS varies more within than between tumor types, and although ECS has a relatively low score on average, more samples need to be studied to see if a group of patients might show association with prognosis or immunotherapy response prediction. For example in breast cancer, patients with the highest 10% of the TIS score had a markedly better prognosis [97]. 

Several studies have demonstrated that EMT contributes to evasion of immune surveillance [98,99,100,101,102,103,104,105]. PD-L1 has a major role in tumor immune escape and also in the development of a permissive immune microenvironment [105]. Different studies have observed an association between PD-L1 expression and mesenchymal characteristics in different tumor types, such as breast, lung, and pancreatic adenocarcinomas, among others. Also, it has been shown that miR-200 targets PD-L1. Moreover, the EMT-TF ZEB1 relieves miR-200 repression of PD-L1 on tumor cells, leading to CD8(+) T-cell immunosuppression and metastasis. 

Regarding carcinosarcomas, PD-L1 expression was significantly higher in lung carcinosarcoma than in conventional non–small-cell lung carcinoma [106], providing a rationale for the potential use of immunotherapy. In this sense, a significant benefit of Nivolumab treatment in PD-L1 positive metastatic pulmonary carcinosarcoma has been reported in some patients [107]. In ovarian carcinosarcoma, PD-L1-positive expression was also observed in about 50% of the tumors, without differences between the epithelial and mesenchymal components [108]. To the best of our knowledge, there are only two studies on PD-L1 expression in ECS. Whereas in one study, PD-L1 was expressed in 25% of the tumors [30], in another, up to 86% of ECS expressed the biomarker [109]. This subset of tumors could benefit from drugs directed to the PD-1/PD-L1 pathway.

## 10. Conclusions and Perspectives

**Figure 5:**
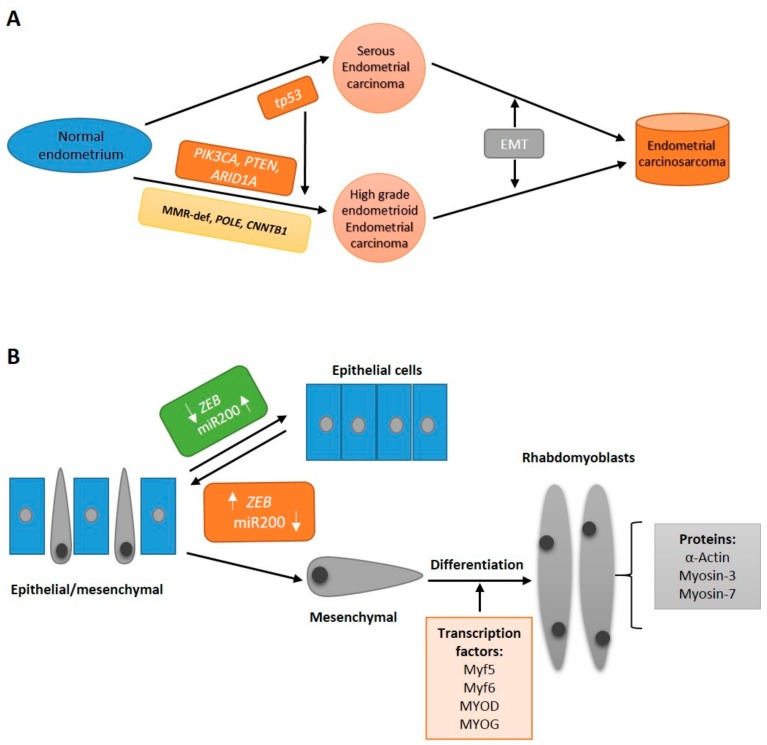
A proposed model of development of endometrial carcinosarcoma. (**A**) Evolution of both endometrioid and serous carcinomas to endometrial carcinosarcoma after eliciting a stable epithelial-to-mesenchymal transition (EMT) program. Transformation of normal endometrium to serous endometrial carcinoma is triggered by mutation in *TP53.* Endometrioid carcinomas with mutations in genes of the phosphatidylinositol 3-kinase (PIK3) pathway or *ARID1A* are more prone to acquire *TP53* mutations than those with mismatch repair deficiency or mutations in *POLE* and *CTNNB1*. (**B**) Endometrial carcinosarcomas are composed by a mixed population of cells representing diverse EMT states. The relative expression of some factors, such as miR-200 or ZEBs, dictate the specific cell state: epithelial, hybrid, or mesenchymal (adapted from Ref. 92).

Carcinosarcoma is a heterogeneous aggressive endometrial carcinoma that probably represents the end-stage of the evolution of both endometrioid and serous carcinomas after triggering a stable EMT program (Figure 5). Molecular observations suggest that, although infrequent, endometrioid carcinomas associated with mutations in *PTEN* or *PIK3CA* are more prone to acquire *TP53* mutations than those associated with MMR-def, *POLE,* or *CTNNB1* mutations. Mutations in *TP53* seem to be essential, but not sufficient, to ECS development, since they are as frequent in ECS as in endometrial serous carcinoma. Although it is not clear what triggers EMT in tumors with *TP53* mutation, a common characteristic of all ECS is the switching of cadherins, the overexpression EMT-TF, and the down-regulation of miR-200 genes. Probably, the crosstalk of different EMT-TF and the differential regulation of miR-200 genes by transcriptional repression or by epigenetic silencing through DNA hypermethylation play a major role in fixing the mesenchymal phenotype. Subsequent activation of specific transcription programs could induce differentiation to diverse mesenchymal tissues.

At present, most patients with ECS are not stratified for treatment according to molecular alterations [110,111]. However, future clinical trials will most likely take into account this data. For example, a recent report has demonstrated the benefit provided when Traztuzumab is included in the treatment of ESC with HER2 amplification [112]. Considering the similarities between ESC and ECS, it is reasonable to think that anti-HER2 therapies would also benefit patients with HER2-positive ECS. Although the relatively low-frequency of ECS hinders efforts to design specific clinical trials, there are promising areas of research, such as the use of immunotherapy in tumors with *POLE* mutations, MMR-def, and high TMB, and also the use of Poly (ADP-ribose) polymerase (PARP) inhibitors in tumors with homologous recombination deficiency, especially due to germline or somatic *BRCA* mutations.

## Figures and Tables

**Table 1:** Comparison of gene mutation frequency among different histological types of endometrial cancer according to The Cancer Genome Atlas Program (TCGA).

GENE	Endometrioid Carcinoma	Serous Carcinoma	Carcinosarcoma
*PTEN*	82%	10%	19%
*PIK3CA*	54%	37%	35%
*PIK3R1*	36%	11%	11%
*CTNNB1*	34%	1%	2%
*ARID1A*	54%	8%	12%
*KRAS*	24%	3%	12%
*CTCF*	31%	2%	7%
*TP53*	21%	88%	91%
*FBXW7*	17%	24%	39%
*PPP2R1A*	11%	38%	28%
*CHD4*	9%	18%	17%
*CCNE1 (ampl.)*	16%	26%	41%
*MYC (ampl.)*	14%	24%	21%
*MECOM (ampl.)*	18%	33%	18%
*PIK3CA (ampl.)*	10%	22%	11%
*ERBB2 (ampl.)*	8%	19%	9%

**Table 2:** Comparison of gene mutation frequency among different series of Endometrial carcinosarcoma (ECS) analyzed by next-generation sequencing.

Gene	Cherniack (n = 57)	McConechy (n = 30)	Jones (n = 361)	Zhao (n = 64) *	Le Gallo (n = 53)
*TP53*	91%	80%	67%	~80%	76%
*FBXW7*	39%	20%		~22%	19%
*PIK3CA*	35%	40%	22%	~20%	34%
*PPP2R1A*	28%	13%		~25%	19%
*PTEN*	19%	27%		~7%	
*CHD4*	17%			~20%	17%
*ARID1A*	12%	10%		~4%	
*KRAS*	12%	10%		~4%	
*PIK3R1*	11%	17%		~4%	
*AKT3*		5%			
*BRCA1*			18%		
*BRCA2*			27%		
*ZFHX3*		7%			
*CSMD3*		23%			
*HIST1H2BJ/G*				21%	
*FOXA2*					15%

* approximated % in a combined series of endometrial and ovarian carcinosarcomas.
